# Concise Review: Mind the Gap: Challenges in Characterizing and Quantifying Cell- and Tissue-Based Therapies for Clinical Translation

**DOI:** 10.1002/stem.416

**Published:** 2010-03-23

**Authors:** Erin A Rayment, David J Williams

**Affiliations:** Healthcare Engineering Research Group, Centre for Biological Engineering, Loughborough UniversityLoughborough, United Kingdom

**Keywords:** Adult stem cells, Cellular therapy, Cell transplantation, Embryonic stem cells, Mesenchymal stem cells, Stem cell transplantation

## Abstract

There are many challenges associated with characterizing and quantifying cells for use in cell- and tissue-based therapies. From a regulatory perspective, these advanced treatments must not only be safe and effective but also be made by high-quality manufacturing processes that allow for on-time delivery of viable products. Although sterility assays can be adapted from conventional bioprocessing, cell- and tissue-based therapies require more stringent safety assessments, especially in relation to use of animal products, immune reaction, and potential instability due to extended culture times. Furthermore, cell manufacturers who plan to use human embryonic stem cells in their therapies need to be particularly stringent in their final purification steps, due to the unrestricted growth potential of these cells. This review summarizes the current issues in characterization and quantification for cell- and tissue-based therapies, dividing these challenges into the regulatory themes of safety, potency, and manufacturing quality. It outlines current assays in use, as well as highlights the limits of many of these product release tests. Mode of action is discussed, with particular reference to in vitro surrogate assays that can be used to provide information to correlate with proposed in vivo patient efficacy. Importantly, this review highlights the requirement for basic research to improve current knowledge on the in vivo fate of these treatments; as well as an improved stakeholder negotiation process to identify the measurement requirements that will ensure the manufacture of the best possible cell- and tissue-based therapies within the shortest timeframe for the most patient benefit.

## INTRODUCTION

Cell- and tissue-based therapies have the potential to treat many conditions where present conventional treatments are inadequate. As such, public expectation remains high that these novel therapies will be “all curing,” even though there have been few completed human trials, and such treatments are currently only available in countries where the regulatory burden is less stringent [1]. In the U.S. alone, there are currently over 750 clinical trials seeking volunteers for studies that include the words “stem cell transplant*” in the intervention [2]. Furthermore, current public policy remains uncertain, as both scientists and regulators strive to set out a clear set of criteria that is appropriate for all aspects of these products. Therefore, there is still a significant gap between promising laboratory-based research and approved final products in this emerging field. Keeping this in mind, this review aims to outline the three main product characteristics—safety, product potency, and manufacturing quality. Next, current measurement precision is analyzed, along with issues of assay sensitivity, detection time, and total cost. Manufacturing challenges are then identified, with an emphasis on regulatory subtleties. Finally, summary perspectives are given on how developers of cell- and tissue-based therapies can move forward in the negotiation processes required in product development.

**Figure 1:**
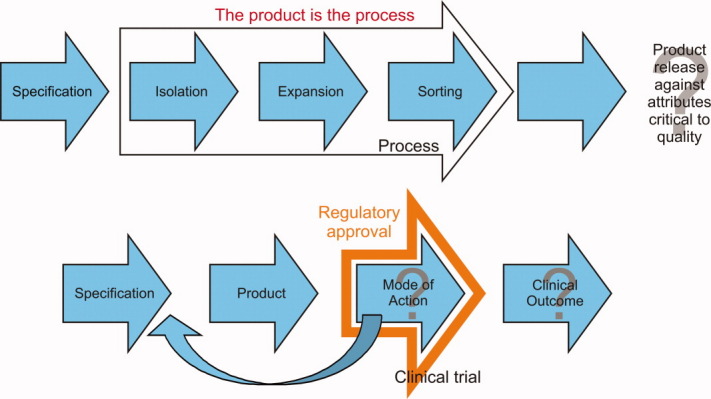
Product design processes for cell- and tissue-based therapies. The top panel outlines the current approach to therapy design, where the process is tightly controlled to maintain high quality. In the bottom panel, an “ideal” product process is outlined, where understanding of the product's mode of action tailors the specifications to provide a beneficial clinical outcome.

In terms of cell- and tissue-based products, both scientists and entrepreneurs are focusing on a number of design and development processes to ensure product success. The current approach to cell- and tissue-based therapy development involves using tightly controlled isolation, expansion, and sorting processes to maintain a high-quality product. In this situation, the required cell specifications are set at the beginning of the process, with specific culture conditions set and then maintained to ensure product consistency. One of the disadvantages of this technique is that the critical quality measurements are still unknown, making it difficult to select appropriate product release tests and optimize clinical efficacy. As our understanding of these advanced products improves, an “ideal” product design process would involve using the product's mode of action to successfully tailor the product specifications to provide a positive clinical outcome (Fig. 1). However, defining a mode of action is complex, and clinical trials are currently the only mechanism of improving understanding of therapeutic action in man. In consequence, there is likely to be frequent iteration of product characteristics before successful clinical outcomes. This redefinition of product requirements and associated challenges with regulatory approval may cause long delays to product launch and provide significant challenges to developers of these advanced therapies.

Once a potential cell- or tissue-based therapy has been identified, all stakeholders in the product development process must be considered to ensure product success. In this review, the stakeholders are broadly divided into three groups: the science base, the entrepreneur, and the public policy actors. The science base includes researchers involved with defining product evidence requirements, whether they are the initial research findings identifying a potential therapy, or the later development work involved with setting product specifications. They are also responsible for collecting the necessary in vitro and in vivo data for product understanding. These can be scientists based at universities and public organizations, as well as those working in both small-to-medium enterprises (SMEs) and larger corporations. Their main motivation is to increase the knowledge base surrounding the advanced therapy. Next, the entrepreneur is the financial force funding these novel therapies. In most cases, the entrepreneur is the leader of an SME, but can also include large pharma, venture capital firms, and government funding bodies. Entrepreneurs, along with investors, are critical in the development of this industry, as they are more likely to fund an promising early-stage technology, whereas large pharma are only likely to become interested on successful completion of phase I trials. The entrepreneur must have a strong business case to support the cell- or tissue-based product, and is most concerned with potential revenues, as well as the creation of a suitable exit strategy for the investor. Finally, the public policy actors are those involved in the clinical aspects of the therapy, and include regulators, reimbursers, clinicians, and patients. For these clinical stakeholders, there is a strong motivation to bring safe, potent, and cost-effective treatments to the general population, with the rate of uptake of these therapies by clinicians and patients strongly influenced by reimbursement. In this stakeholder group, professional clinical bodies and patient advocacy groups can also influence therapy development and approval through public awareness campaigns and government lobbying.

## PRODUCT SAFETY

In terms of the product itself, safety is the primary concern for regulatory agencies when examining potential new treatments. Following this, ensuring sterility is a priority for cell- and tissue-based therapy manufacturers. Current Food and Drug Administration (FDA) guidelines outline the requirements for microbiological testing of aerobic and anaerobic bacteria and fungi (21 CFR 610.12). Since viable cell- and tissue-based products cannot undergo a terminal sterilization step, as is the case with other pharmaceuticals, they need to be manufactured under aseptic conditions [3]. In addition, these products are likely to have a short shelf-life, which often means that these products are administered to patients before current sterility test results are available [4]. Because of these obvious issues, there is a strong need to develop, validate, and implement faster testing techniques than those currently accepted.

In terms of mycoplasma, the current testing standard for mycoplasma contamination uses a broth and agar technique, which simply analyses bacterial growth on nutrient media, as described in the US 21 CFR 610.30. However, due to the time constraints related to a short shelf-life, faster techniques are being considered, for example, rapid microbiological methods. This technique would provide the same performance standards as are currently used in 21 CFR 610.30, but would also provide these results in a much shorter time period, that is, from weeks to hours [4]. Investigators can also choose to validate polymerase chain reaction (PCR)-based mycoplasma detection systems according to the guidelines of the FDA and European Pharmacopoeias. Along with current PCR test kits, Cambrex (East Rutherford, NJ, http://www.cambrex.com/) currently produces a MycoAlert Mycoplasma Detection Kit that claims to be able to detect <50 colony-forming units per milliliter of mycoplasma, as well as providing results in less than an hour [5]. Considering the current 2- to 3-week wait for the traditional broth and agar technique, this is a considerable improvement.

Viruses are another form of contamination that have to be tested for before a product can be released. In terms of possible donor contamination, a donor blood sample can be analyzed for the presence of human immunodeficiency virus, hepatitis B and C viruses, and human T-lymphotropic virus [6]. However, there is also the possibility of viral contamination from reagents involved in the cell culture process, for example, fetal calf sera (FCS) and porcine trypsin [7,8]. It is also important to note that many viruses have extended latency periods, which means that a negative result at the time of culture does not necessarily eliminate all patient risks [9]. In regards to transmissible spongiform encephalopathies, there is manufacturing guidance regarding how to deal with possible risk products [3,10]. Specifically, there must be a constant traceability of animals, geographical limits on animals to countries classified as geographical bovine spongiform encephalopathy (BSE)-risk I and II [11], as well as specific animal stunning methods [10].

Endotoxin or pyrogenicity testing is another requirement of all parenteral drugs, biological products, cell- and tissue-based therapies, and medical devices. This can be measured by using one of two tests, either the rabbit pyrogen test (USP151) or the limulus amebocyte lysate assay (USP85) [12–14]. Validation of the individual test method must be established (21 CFR 610.13), including whether the cell preparation can interfere with the test selected [15]. Of note, it is important to remember that some substances that have passed these tests have still been shown to be pyrogenic in human patients. Therefore, some suggest culturing in vitro human blood mononuclear cells with the final product, and injecting this cell culture fluid into rabbits to determine species-specific pyrogenicity [16]. In terms of large-scale culture, there are currently high throughput, FDA-licensed, rapid endotoxin testing devices available, for example, Endosafe-MCS (Charles River Laboratories, Wilmington, MA, http://www.criver.com/), which have been used in conventional bioprocessing applications.

There are many contributing factors that affect cellular proliferation and survival. All cell culture conditions are extremely important in maintaining large numbers of viable cells [17]. Culture media can often be a difficult choice, as the majority available contains some animal products that may increase the risk of disease transmission to the patient, with various current good manufacturing practice (cGMP) approved media in the literature [18–20]. However, all of these preparations contain FCS, which is an animal-based product that could potentially transfer disease, or even cause immunogenic reactions in the patient [21,22]. One study has reported that patients receiving a hematopoietic stem cell transplantation displayed antifetal calf serum antibodies, which have also been found in normal human populations [23]. Although they did not find alloantibodies as a result of sensitization to the mesenchymal stromal cells [23], it is important to try to reduce any potential patient risks by avoiding the use of non-human products in culture. Therefore, autologous patient serum has been considered as an FCS replacement [24]. However, in vitro studies have shown that sera from aged patients can also inhibit cellular function [21,25], with young sera enhancing cell proliferation [26].

Knowledge regarding the body's immune response to cell- and tissue-based products will also influence the safety of the product. If these products are recognized by the host as “foreign,” the risk of rejection and associated pathologies may be greater than the potential benefit to the patient [27]. In addition, human leukocyte antigen (HLA) matching may be important in pairing donors to hosts, since fewer HLA mismatches may lead to better tissue acceptance [27,28]. However, unlike organ transplantations, not all cell- and tissue-based therapies may be treating life-threatening conditions, which makes the use of immune suppression drugs that may promote infection much more difficult to justify [29]. Therefore, the potential immune-privileged state of human embryonic stem cells (hESCs) has been discussed as a way to overcome these problems [29]. However, Drukker et al. have shown that although major histocompatibility complex-I expression is low in hESCs, it can be rapidly induced, which could lead to rejection [30]. As a result, there are still many issues surrounding host immune response that have to be considered.

Since large number of cells are generally required for administration, there is often a need to perform extensive in vitro culturing over large periods [24]. However, this extended amplification time can also lead to cellular senescence, as well as genetic and epigenetic changes [21,24]. Karyotype testing should be performed once cells have been maintained in culture for significant periods to ensure chromosomal stability [31]. This can be achieved by counting a minimum number of cells in a metaphase spread (>20) that has been analyzed using Giemsa staining [32,33]. If aneuploidies are detected, the sample is immediately discarded removing the need for further analysis. However, it is important to note that standard genetic analysis only identifies gross chromosomal abnormalities, and can miss submicroscopic DNA alterations that can potentially affect cell phenotype [34]. Furthermore, there is very little knowledge about how these abnormalities might be clinically significant or their potential risk to the patient. In terms of epigenetic aberrations, the possibility of irregular cell growth and potentially cancer is always a major concern [24]. For treatments involving the use of hESCs, the risk of undifferentiated cells contaminating the eventual therapy is ever present. As a result, many authors suggest that all undifferentiated and unwanted cells will need to be removed before patient administration [35,36].

## PRODUCT POTENCY

Potency is defined by the FDA as “the specific ability or capacity of the product, as indicated by appropriate laboratory tests or by adequately controlled clinical data obtained through the administration of the product in the manner intended, to effect a given result” [37]. Efficacy generally refers to the ability of a drug or medicinal treatment to cause a functional response in the patient, and is proportional to the potency of the therapy. Effective tests to determine product potency will be required to ensure a cell- or tissue-based product is manufactured to the same consistent standards [37]. With this in mind, proper characterization and understanding of cell function, or mode of action, is the most important factor in determining whether a cell- or tissue-based therapy will function effectively in vivo. However, as complete characterization of some cell processes are still unknown, it is very difficult to accurately predict every consequence of a particular cell once placed within a patient.

**Figure 2:**
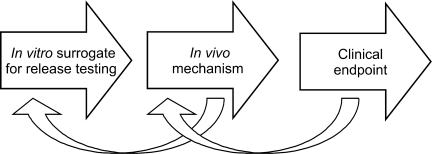
Dynamic feedback process for determining effective in vitro surrogates for release testing. As the eventual fate of the cell once implanted is still unknown, continual feedback will be needed to improve cell- and tissue-based therapy product design from the original bench-based development through clinical trials and eventual postmarketing surveillance.

In terms of cell- and tissue-based products, unlike more established pharmaceuticals, there will be continual feedback to help inform the selection of in vitro efficacy tests, as the eventual fate of the cell once administered is still unknown. As a result, initial release assays will be required to be based on well understood in vitro data collected during cell characterization and development. Preclinical safety studies and nonclinical testing are also likely to provide further information on possible in vivo actions of the product. Furthermore, regulatory authorities require such in vivo testing to identify safe dosages in humans, potential target organs for toxicity, and safety parameters for clinical monitoring [38]. Although limited to the beginning of clinical development, these nonclinical animal in vivo studies should also be sufficient to identify potential adverse affects that might then occur in the first in man clinical trial [39]. Leading on from this, once manufacturers have approval to conduct clinical trials and can correlate this data with in vitro product specifications, improved product release tests will be designed and integrated into the product development process. In addition, once the product has been approved and used by a larger population, postmarketing surveillance will provide valuable insights into the product mechanism. Therefore, clinical results will provide expanded knowledge on in vivo mechanisms, with the improved understanding of these mechanisms allowing for better design of in vitro release tests, as shown in Figure 2. However, these product release test improvements are also going to be influenced by the resulting regulatory implications.

Efficacy tests should always be cell-specific, and ideally, test the function of the cell that will be required in an in vivo situation. For example, embryonic stem cell-derived cardiomyocytes can be tested for function by analyzing spontaneous beating of embryoid bodies in culture, or even by analyzing levels of functional adrenoreceptors [40]. Preclinical in vivo testing can provide enhanced product understanding and demonstrate initial proof of concept, if issues such as the use of a relevant animal species, age, physiological state, manner of delivery, and stability of the test material are all carefully considered [38]. However, in some cases, in vitro assays can even be more sensitive and give more useful data than in vivo trials [41], especially when considering such issues as expense, variability, and time periods required for results [42]. As a result, there are many issues concerning the “best” way to test for product potency before reaching clinical trials, especially in terms of whether in vitro efficacy tests will act as successful surrogates for predicting in vivo human clinical response.

Clinical endpoints have to be defined at an early-stage to allow for proper evaluation of cell- and tissue-based therapies in patient trials. For example, for cardiac applications, Losordo et al. used bioactivity measurements to assess the efficacy of their CD34+ cell suspensions [43]. They evaluated angina frequency, nitroglycerine use, exercise tolerance, single-photon emission computed topography imaging, and quality of life testing [43]. Other groups have used enhanced myocardial contractility and left ventricular ejection fractions, both in relation to normal limits, as suitable endpoints for their cardiac applications [44]. However, due to the complexity of several clinical applications, optimal efficacy measurements may evolve over time due to improved clinical information becoming available to inform the decision.

## PRODUCT QUALITY THROUGH CONTROLLED AND AUDITABLE MANUFACTURING PROCESSES

### Cell Number and Viability

Once the manufacturer is certain that the product is safe, the next step is to produce sufficient quantities of cells to be therapeutically effective in a patient. In addition, although the number of cells produced is extremely important, the cell viability itself is the primary concern for determining the actual cellular effect in the body. This can be measured using various assays, such as using trypan blue and a hemocytometer, as well as more sophisticated measures of cell metabolic activity [45]—with both providing quantitative data. Techniques such as flow cytometry [46] and multiplex assays [47] allow several parameters to be measured at the same time. For large-scale manufacture, automated cell counters, such as the Cedex (Innovartis, Bielefeld, Germany, http://www.innovatis.com/), provide increased cell count and viability accuracy, as well as large time savings over traditional manual methods [48]. However, most of these viability percentages simply measure how many cells are “alive,” not how many cells are actively metabolizing and playing a productive role in their environment.

Cryopreservation is a further point to consider when setting cell number and viability specifications for cell- and tissue-based products. Because of the inherent limitations of storing and transporting actively growing cells, most product developers aim to cryopreserve their product to allow for long-term product storage until the therapy is needed for patient administration. Currently, the two main methods of preparing cells for cryopreservation include slow-freezing with a permeating cryoprotectant or vitrification, which involves partial replacement of the water content of the cells with a cryoprotectant and then rapid cooling by immersion in liquid nitrogen [49]. The main problems associated with cryopreservation include loss of cell numbers, decreased cell viability, and slower growth rates on recovery. In addition, few cryopreservation protocols appear to work well for hESCs [50]. The poor recovery rates post-thaw can then lead to extended culture times, which have the possibility of exerting selective pressure on the cells and consequently expanding different hESC subpopulations in different laboratories [50]. Therefore, it appears that more understanding of the effects of cryopreservation, as well as a standardized protocol specific to hESCs, is urgently needed.

### Cell Phenotype, Function, and Mode of Action

Cell phenotype is generally characterized through flow cytometry or fluorescence-activated cell sorting, which can analyze a cell's stage of proliferation, differentiation, or activation through cell surface and intracellular markers [51]. These markers are commonly used to identify individual cell populations in heterogeneous samples. In terms of hESCs, Siti-Ismail et al. state that they should be Oct-4+, Nanog+, stage-specific embryonic antigen-4+, tumor rejection antigen (TRA)-1-60+, and TRA-1-81+ to maintain their “stemness” [52]. However, although biomarkers may be useful in distinguishing different cell phenotypes, they do not always provide a correlation to cell function. One example of this is that patients with chronic ischemic cardiomyopathy have a similar number of endothelial progenitor cells to healthy volunteers, but their colony-forming ability is significantly impaired [53]. Therefore, in terms of cell- and tissue-based therapies, how the cells act in the body might be more important than their immunophenotype in vitro.

Cellular morphology is also used to analyze cell populations using various microscopy techniques to determine whether cells appear true to their phenotype. For example, fibroblasts are commonly characterized by their elongated spindle-shaped appearance when cultured in vitro [54]. In addition, various stages of cellular senescence can be identified through microscopy, which can be seen visually as enlarged nuclei and multinucleated cells following extensive culture of human epithelial cells [55]. There are several microscopy techniques available to analyze cellular morphology, from fluorescent confocal microscopy to widefield live cell imaging [56]. Many companies are currently developing high throughput and high-content screening platforms for automated analysis of cellular morphology, intracellular localization, and cellular dynamics [56]. For example, Carl Zeiss MicroImaging has a Cell Observer system that can acquire up to 300 images/second, displays a resolution of 12 megapixels, and has a signal to noise ratio of at least 1:2,500 [57]. However, these automated systems still require the user to define their own parameters and analyze the images accordingly.

## HOW PRECISELY CAN WE TAKE THESE MEASUREMENTS?

Existing quantification techniques for measuring safety parameters such as bacterial and fungal load, virus contamination, and endotoxin levels appear to be sensitive enough to prevent adverse patient events. Similarly, genetic testing is currently precise enough to identify known gross chromosomal abnormalities, assuming that a sufficient minimum number of metaphase spreads is counted [32]. However, the main drawbacks of the current techniques are that they require long incubation times and can be costly. Therefore, improvements in the processing times, reduction in costs, and conversion to automated methods may improve the robustness of current contamination detection methods. Furthermore, the main concern regarding safety measurements is the current inability to remove all unwanted cells. For this to occur, current cell detection methods will need to be sensitive enough to detect as few as tens of cells in a large-cell suspension, for example, <0.00001%. Considering that current advanced cell sorters claim to have only a sensitivity limit of 98% [58], there need to be significant advances in this technology to ensure patient safety. However, the multiple purification steps required to ensure a sufficient standard of quality may add several costs on to the product, in terms of reagents, work hours, and also initial cell numbers required to give a purified product.

To meet product specifications, cell number and cell viability measurements must be accurate so that specific product dose can be determined. The accuracy of cell counts can also be quite variable, with Brinkmann et al. reporting a 20% counting error for manual cell counts, with only a 5% counting error for the Cedex platform [59]. Therefore, although automated cell counting systems have several advantages over manual counting, improvements still need to be made. Of note, the final product acceptance range should be carefully considered as the limitations of the machine must be taken into account. This may include the tolerancing of machine specifications, as well as factoring in measurement system errors that may contribute to misleading data. An extremely narrow range might cause products to be rejected due to these inaccuracies rather than actual product failure. If an out-of-specification (OOS) result has occurred, FDA regulations require a thorough, timely, unbiased, well-documented, and scientifically sound investigation—even if the batch has been rejected [60]. For products covered under approved full and abbreviated drug applications, a field alert report (FAR) must be submitted within three working days of a OOS test result, with a follow-up FAR submitted once the OOS investigation is finalized [60]. Complementary to this procedure, the manufacturer needs to implement a corrective and preventive action system to deal with complaints, product rejections, nonconformances, recalls, deviations, audits, regulatory inspections, and findings, as well as process and product quality trends [61]. This system should be proportionate to the level of risk and in line with ICH Q9, resulting in improvements in both the product and process, along with enhanced product and process understanding [61].

## MANUFACTURING CHALLENGES

Issues concerning safety highlight the importance of manufacturers being able to maintain stringent compliance to cGMP standards, as well as clear definition of these standards by the appropriate regulatory authorities. However, once safety has been addressed, the current biggest challenge for manufacturers of cell- and tissue-based therapies is in the development of representative potency assays to evaluate the final product. According to a recent FDA guidance document, potency assays must be specific, quantitative, meet predefined criteria, include appropriate standards and controls, be fully validated and measure both identity and strength of all active ingredients [37]. Therefore, due to the inherent heterogeneity in the cell population, these requirements can only be met if the product is fully defined and manufactured to the same consistent standards. This consistency will rely on strong quality systems controlling both the product and the manufacturing process itself. One such quality system is quality by design, which focuses on building quality into the product through a thorough understanding of the process, combined with a clear knowledge of manufacturing risks and mitigation strategies [62]. This system can aid manufacturers by reducing time to approvals, but can also build significant cost into the manufacturing process through the shear amount of testing required throughout production.

Product developers must also clearly identify when their product release tests will take place, as specifications set for products pre- or postcryopreservation may vary significantly and therefore prompt costly batch failures if not clearly defined. In most cases where cells are the final product, manufacturers will establish a two-tier system of a master cell bank (MCB) and a working cell bank to ensure consistency of the final product. The MCB must be fully validated and include the following information: the cell origin, standard operating procedures for all manipulations, genetic and/or phenotypic marker characterization, sterility testing, expiration dating, and complete testing of thawed/expanded cells [15]. However, due to the potentially small cell numbers available, alternative samples to the final product may sometimes be used to minimize product loss.

In terms of stability, the FDA has clear guidelines on testing that must be performed to gain product approval. Although these guidelines were originally developed for biotechnological products, for example, proteins, they can be adapted to include cell- and tissue-based products. In terms of long-term cell storage and batch testing, manufacturers must provide stability data on at least three batches that have undergone manufacturing, but have not yet entered the formulation stage, with a minimum of 6 months data for products requiring long-term storage [63]. In addition, stability information should also be provided for at least three batches of the drug product (manufactured in a manner that is representative of that used at scale). Finally, the manufacturer must generate a stability-indicating profile that takes into account changes in identity, purity, and potency of the final product [63].

Together with contamination, eliminating the potential for cross-contamination is a major issue for manufacturers of cell- and tissue-based therapies, especially if multiple autologous therapies are processed in the same laboratory at the same time. Cell line cross-contamination has long been a concern of those working in the area, with recent reports estimating cross-contamination levels of commercially available cell lines between 18% and 36% [64]. The segregation of individual cell types, whether cell lines or individual patient cells, is pivotal to maintaining quality of cell- and tissue-based products. However, in terms of manufacturing multiple autologous therapies concurrently, the cost of complete segregation of patients' cells in dedicated incubators and biosafety cabinets may make the cost of such therapies prohibitive for many patients. Therefore, there are many distinct challenges to manufacturers of autologous therapies, compared with those who might only handle one distinct cell type for allogeneic treatments.

## CONCLUSIONS

**Figure 3:**
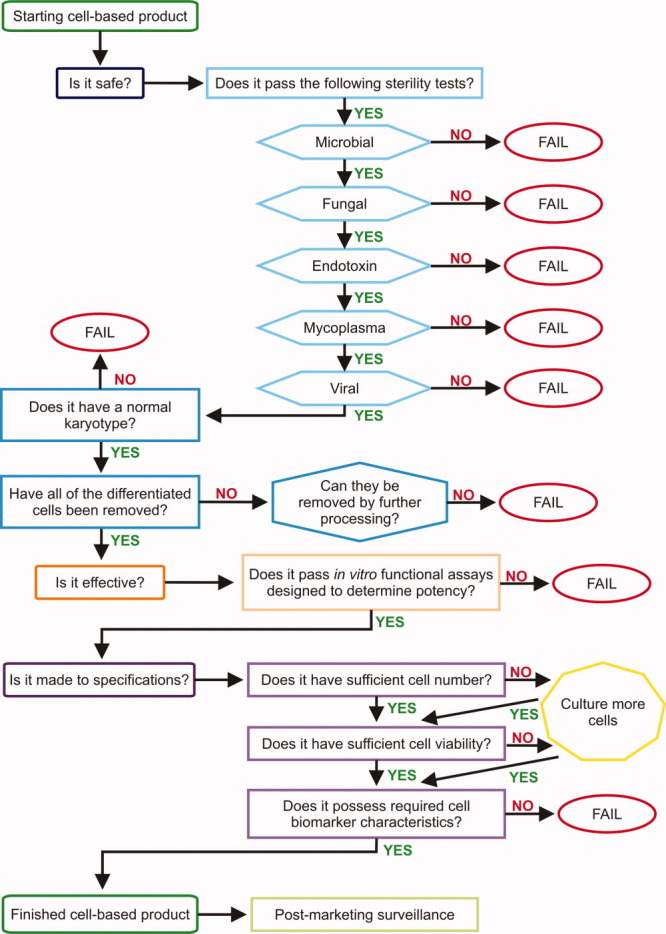
General test process for cell- and tissue-based therapies. This process is divided into the three main stages of development, including safety, efficacy, and purity of manufacture. In each stage, there are a number of requirements that must be met to allow the product to continue through the next stage of the process.

As shown in this review, there are many issues to consider when preparing a cell- and tissue-based therapy for commercial use, with a general product process outlined in Figure 3. This flowchart includes a series of steps that must be completed before the product can be approved for human use. Initially, safety testing is critical, including assays for potential microbial, fungal, endotoxin, mycoplasma, and viral contamination; karyotype testing; and enrichment for the required cell population. Once safety has been established, the product must pass in vitro functional assays designed to act as surrogate measures for clinical effectiveness [65]. These potency assays must be fully validated to meet regulatory requirements, including appropriate standards and controls. The third stage of this process analyses whether the product has been made to a certain set of specifications, and therefore ensures the high quality of the process and the product. This generally entails cell number and viability measurements, along with cellular biomarkers that can act as identifiers of cell type and purity. Once all of these categories have been addressed the product should be suitable for human use, with continual postmarketing surveillance after product launch to collect valuable information that may further improve this process.

**Figure 4:**
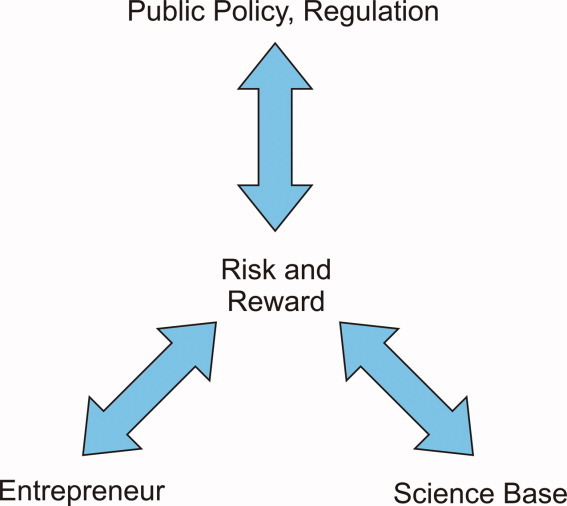
Product development negotiations. Entrepreneurs and public policy actors have different goals, each required to manage risk, reward, and time to market. Management of risk/reward tradeoffs requires both negotiation and consensus on the evidence requirements for product adoption. The science base has a critical role in defining the evidence requirements and collecting necessary data.

Depending on your perspective, all stakeholders have different requirements for these novel therapies. These potentially conflicting perspectives and motivations will be difficult to condense into a singular solution that manages the risks, rewards, and time to market for each treatment. In terms of rewards, these can be returns to the entrepreneur, medical benefits to the patient, as well as financial savings for the healthcare providers, whether government or private organizations. Convergence on the risk/reward tradeoffs requires both negotiation and a consensus on the evidence requirements for product adoption. In this situation, the science base has a critical role in assisting both the definition of the evidence requirements for the regulators, as well as working with clinicians to collect the necessary data to demonstrate in vivo effectiveness. Hence, all of these needs will have to be balanced according to the stakeholder's influence on getting the product to market (Fig. 4). Although scientific input is a key mechanism for supplying robust data, commercial funding is likely to be the primary determining factor in the development of a successful product. In turn, a product can only be available to the public after regulatory approval, which means that public policy concerns regarding safety need to have priority, followed by cost-effectiveness. Clinician and patient needs are also important, but as long as consultation is carried out during product development, any concerns should be addressed, at least to a certain extent, before full market release. This negotiated approach to product development should allow for all opinions to be noted, and then decisions based on the stakeholder priority.

One major problem is that there are no current procedures and scarce stakeholder resources for these negotiations. Therefore, it appears that a common process will need to be developed to pool existing data and identify gaps in the information required for informed discussion. Furthermore, as there is still great debate over what measurements should be used to confirm these advanced therapies are safe and effective; scientists need better resources to generate improved knowledge on the in vivo fate of these treatments to influence measurement selection. Therefore, as more in vivo data is gathered, and safety profiles become easier to assess, product efficiency should improve greatly from first- to second- to third-generation products. Hopefully, the clinician and the patient will be willing to wait for these more effective cell- and tissue-based therapies.
